# Bridging Genomics to Cardiology Clinical Practice: Artificial Intelligence in Optimizing Polygenic Risk Scores: A Systematic Review

**DOI:** 10.1016/j.jacadv.2025.101803

**Published:** 2025-06-25

**Authors:** Kaveh Hosseini, Nazanin Anaraki, Parham Dastjerdi, Sina Kazemian, Mandana Hasanzad, Mohamad Alkhouli, Mahboob Alam, Khurram Nasir, Jamal S. Rana, Ami B. Bhatt

**Affiliations:** a Tehran Heart Center, Cardiovascular Diseases Research Institute, Tehran University of Medical Sciences, Tehran, Iran; b Personalized Medicine Research Center, Endocrinology and Metabolism Clinical Sciences Institute, Tehran University of Medical Sciences, Tehran, Iran; c Department of Cardiovascular Medicine, Mayo Clinic, Rochester, Minnesota, USA; d Division of Cardiology, Baylor College of Medicine, Houston, Texas, USA; e Division of Cardiovascular Prevention and Wellness, Department of Cardiology, Houston Methodist DeBakey Heart & Vascular Center, Houston, Texas, USA; f The Permanente Medical Group, Department of Cardiology, Oakland Medical Center, Oakland, California, USA; g Chief Innovation Officer, American College of Cardiology, Associate Professor, Harvard Medical School, Washington, District of Columbia, USA; h Heart Center, Massachusetts General Hospital, Harvard Medical School, Boston, Massachusetts, USA

**Keywords:** artificial intelligence, machine learning, polygenic risk scores, precision medicine, risk prediction

## Abstract

Despite advances in cardiovascular disease risk stratification, traditional risk prediction models often fail to identify high-risk individuals before adverse events occur, underscoring the need for more precise tools. Polygenic risk scores (PRS) quantify genetic susceptibility by aggregating genetic variants but face challenges in practice. This systematic review investigates how artificial intelligence (AI) and machine learning algorithms can optimize PRS (AI-optimized PRS) to improve cardiovascular disease prediction. Analyzing 13 studies, we found that AI-optimized PRS models enhance predictive accuracy by improving feature selection, handling high-dimensional data, and integrating diverse variables—including clinical risk factors, biomarkers, imaging, and combining multiple PRS. These models outperform nonoptimized PRS models, providing a more comprehensive understanding of individual risk profiles. Evidence suggests that AI-optimized PRS can better stratify patients and guide personalized prevention strategies. Future research is needed to explore sex differences, include diverse populations, integrate AI-optimized PRS into electronic health records, and assess cost-effectiveness.

## Highlights

• AI-optimized polygenic risk scores show promising potential for improving early detection and personalized prevention of cardiovascular diseases.
• By integrating genetic and clinical data, AI-optimized PRS enhance conventional models in predicting cardiovascular disease outcomes.
• The incorporation of clinical risk factors, biomarkers, and imaging data into AI-optimized PRS significantly enhances their predictive accuracy and robustness.
• Further research is needed to expand AI-optimized polygenic risk scores to diverse populations and integrate them into electronic health records.

Cardiovascular disease (CVD) remains a leading global cause of morbidity and mortality, highlighting the urgent need for effective early diagnosis and preventive strategies.^1^ Coronary artery disease (CAD) is a common form of CVD and significantly contributes to the global burden of CVD.^2^ Traditional risk prediction models for CVD are often based on demographics and clinical features, including age, sex, lipid levels, blood pressure, and diabetes, to estimate an individual’s 10-year CVD risk.^3^ Despite decades of advancements, these tools often struggle to identify high-risk individuals. In particular, they are prone to underestimating or overestimating risk, especially in individuals at the margins of established risk categories. Furthermore, these models frequently underperform in non-White self-identified racial and ethnic groups, largely due to their development in predominantly European ancestry populations.^4^ Consequently, many people remain without essential preventive measures until after experiencing a serious event, such as a myocardial infarction or stroke.^5^

Given the polygenic nature of CVD, polygenic risk scores (PRS) can offer a powerful approach to identifying individuals at elevated risk of CVD before clinical symptoms emerge. Integrating genetic data into risk prediction models has shown promise for enhancing early detection and personalized prevention.^6^ PRS offer a comprehensive method to quantify genetic susceptibility by aggregating the effects of numerous single nucleotide polymorphisms (SNPs) associated with disease risk.^6^^,^^7^ Constructed using genome-wide association studies, PRS reflect the cumulative impact of genetic variants, each contributing a small effect size, to generate a comprehensive genetic risk profile.^8^ For CAD, recent PRS models incorporated hundreds of thousands to over a million common single nucleotide variants, capturing the polygenic nature of genetic susceptibility.^9^ This expanded variant inclusion underscores the potential of PRS to refine risk stratification and improve predictive accuracy beyond traditional clinical risk factors.^10^

However, the clinical implementation of PRS faces several significant challenges that must be addressed to realize their potential in precision medicine. One major limitation is the need to account for linkage disequilibrium—the nonrandom association of alleles at 2 or more genetic loci within a population—which is essential for accurately modeling genetic contributions to disease risk. Additionally, integrating genetic data with clinical variables poses substantial complexity, requiring methods capable of managing high-dimensional data sets and capturing nonlinear interactions between genetic and environmental factors.^11^ Traditional statistical methods can incorporate genetic data into clinical models but often struggle to address intricate patterns and diverse data types, which limits the effectiveness of PRS integration.^12^

To address these challenges, artificial intelligence (AI) and machine learning (ML) algorithms offer a transformative solution for optimizing PRS. ML algorithms enhance feature selection, manage high-dimensional data, and integrate diverse data sets—ranging from biomarkers and imaging data to multiple PRS—to improve predictive accuracy.^13^ Furthermore, AI-optimized models facilitate clinical adoption by incorporating genetic data into systems such as electronic health records (EHRs) for real-time monitoring and personalized interventions. These advancements underscore the potential of AI-driven approaches to enhance the clinical utility of PRS. In this systematic review, we aim to summarize the current evidence on the role of AI and ML algorithms in optimizing PRS for improved CVD prediction and risk stratification.

## Methods

This study follows the guidelines established by the Preferred Reporting Items for Systematic Reviews and Meta-Analyses (PRISMA) protocols.^14^ The study protocol has been registered in the International Prospective Register of Systematic Reviews (PROSPERO) under the registration number CRD42024596037. As this systematic review is based on previously published studies and utilizes only published summary data, no additional ethical approval was required.

### Search strategy and data extraction

We conducted a systematic search of PubMed, Embase, Scopus, and the Cochrane Library databases from their inception to December 15, 2024. The search strategy included the keywords “PRS” AND “CVD” AND (“AI” OR “ML”), along with their equivalent terms, without any restrictions on study type or language. Detailed search strategies for each database are provided in Supplemental Appendix. Studies were included if they have employed ML algorithms to develop a PRS through feature selection or if they utilized ML to integrate various data sources—such as biomarkers, imaging data, clinical risk factors, or multiple PRS—for predicting CVD outcomes. Studies were excluded if they did not apply ML algorithms to develop or optimize a PRS model, as well as those that considered CVD risk factors—such as diabetes, hypertension, or hypercholesterolemia—as primary model outcomes. Moreover, case reports, conference abstracts, reviews, and studies lacking relevant outcome data were excluded.

After removing duplicates, 2 reviewers (N.A. and P.D.) independently screened the titles and abstracts based on the eligibility criteria, resolving any discrepancies through mutual consensus. The full texts of eligible studies were reviewed, and the following prespecified variables were extracted: study year, type of PRS (previously developed or original), PRS database, study population, ML algorithm, outcome, AI-optimized PRS components, presence of external validation, comparison metrics, and main findings.

### Quality assessment

The quality of the included studies was independently evaluated by 2 reviewers (N.A. and P.D.) using the Quality Assessment of Diagnostic Accuracy Studies-2 (QUADAS-2) tool, with disagreements resolved through consultation with a third reviewer (S.K.). QUADAS-2 assesses the risk of bias and applicability across 4 domains: patient selection, index test, reference standard, and flow and timing.^15^ In summary, the patient selection domain evaluates potential biases in recruitment and exclusion criteria to ensure that the study population is representative. The index test and reference standard domains address biases related to how these PRS analyses were conducted and interpreted. The flow and timing domain examines the time interval between the index test and reference standard, as well as whether all participants underwent the same reference standard. This rigorous quality assessment ensured that our review provides a reliable synthesis of the current evidence on the role of AI and ML algorithms in optimizing PRS for CVD risk prediction.

## Results

**Figure 1:**
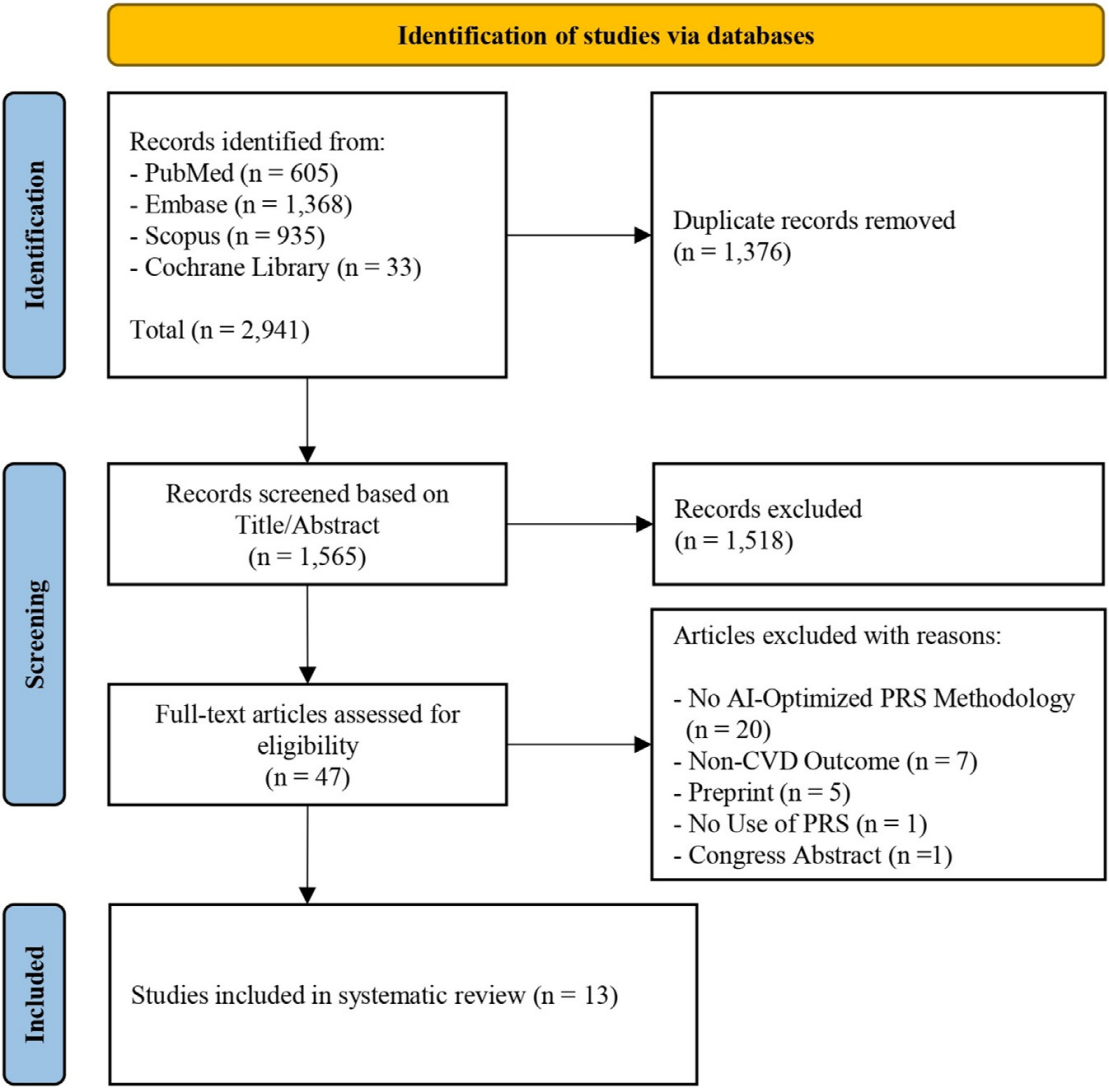
The PRISMA Flow Chart of Study Screening and Selection AI = artificial intelligence; CVD = cardiovascular disease; PRISMA = Preferred Reporting Items for Systematic Reviews and Meta-Analyses; PRS = polygenic risk score.

Our initial systematic search identified 2,941 studies. After removing duplicates, 47 studies were deemed eligible for full-text screening, and 13 studies were ultimately included in the systematic review based on the inclusion criteria (Figure 1).^11^^,^^16^, ^17^, ^18^, ^19^, ^20^, ^21^, ^22^, ^23^, ^24^, ^25^, ^26^, ^27^ These studies were published between 2021 and 2024. They utilized genetic data from large-scale biobanks, including the UK Biobank (11 studies),^11^^,^^16^, ^17^, ^18^, ^19^, ^20^, ^21^^,^^23^^,^^25^, ^26^, ^27^ multiple United States biobanks (3 studies: BioMe, atherosclerosis risk in communities study, Mayo Clinic, and Penn biobanks),^19^^,^^23^^,^^25^ China biobanks (2 studies: China-PAR and China Kadoorie biobanks),^16^^,^^24^ FinnGen (1 study),^21^ and Dan-NICAD (1 study).^22^ CAD was the primary outcome in most studies (84.6%), while 2 studies considered MI and major adverse cardiovascular events as their primary endpoints.^25^^,^^26^ Notably, external validation was conducted in only 5 studies (38.5%).^16^^,^^19^^,^^21^^,^^23^^,^^25^ The PRS in these studies were generated using a wide range of genetic variants, ranging from 50 to 1.7 million SNPs (Table 1).

**Table 1:** Study Characteristics, Machine Learning Models, and External Validation of Included Studies

First Author, Year	Type of PRS	PRS Database	Machine Learning Model	External Validation	Outcome
Naderian et al, 2024^11^	PGS000018 (CHD)	UK Biobank (general population)	Elastic net regression (feature selection+classification of polysocial score & AI-optimized PRS models) XGBoost (feature selection+classification of polysocial score & AI-optimized PRS models)	No	10-y CAD (MI, coronary revascularization)
Mazidi et al, 2024^16^	New PRS	Training and validation: China Kadoorie Biobank External validation: UK Biobank (general population)	Lasso regression (feature selection for conventional & AI-optimized PRS models) Boruta (feature selection+classification for proteomic models)	Yes	CAD
Alireza et al, 2024^17^	New PRS	UK Biobank (general population)	Lasso regression, support vector machine, random forest (feature selection for conventional & AI-optimized PRS models) Maximum relevance minimum redundancy, random forest (classification for conventional & AI-optimized PRS models)	No	CAD
Sng et al, 2024^18^	New PRS	UK Biobank (general population)	Random forest (feature selection+classification for conventional & AI-optimized PRS models)	No	CAD
Forrest et al, 2023^19^	New PRS	Training and validation: BioMe Biobank External validation: UK Biobank (General population)	Random forest (feature selection+classification for conventional & AI-optimized PRS models)	Yes	CAD
Klau et al, 2023^20^	New PRS	UK Biobank (general population)	Ridge regression, random forest, deep neural network (feature selection+classification for integrating multiple PRS)	No	CAD
Lin et al, 2023^21^	New PRS	Training and validation: UK Biobank (general population) External validation: FinnGen Biobank (exclusion of angina-only cases)	Elastic net regression (feature selection+classification of conventional PRS models)	Yes	CAD
Møller et al, 2023^22^	New PRS	Dan-NICAD Biobank (patients with symptoms suggestive of CAD)	Elastic net regression (feature selection+classification for AI-optimized PRS model)	No	CAD
Norland et al, 2023^23^	New PRS	Training and validation: UK Biobank & Atherosclerosis Risk in Communities Biobank External validation: Mayo Clinic (general population)	Lasso regression (feature selection+classification for 2 AI-optimized PRS models)	Yes	CAD
Lu et al, 2022^24^	New PRS	Training set: General population from Fuwai Hospital, National Center for Cardiovascular Diseases, China. Validation cohorts: China-PAR Biobanks (InterASIA, ChinaMUCA-1998, and CIMIC (no history of CVD)	Elastic net regression (feature selection+classification for AI-optimized PRS model)	No	CAD
Nam et al, 2022^25^	New PRS	Training and validation: UK Biobank External validation: Penn Medicine Biobank (general population)	Graph-based semisupervised learning (feature selection+classification for network PRS model)	Yes	MI
Steinfeldt et al, 2022^26^	PGS000011 (CAD) PGS000018 (CAD) PGS000057 (CAD) PGS000058 (CAD) PGS000059 (CAD) PGS0000398 (stroke)	UK Biobank (no history of CVD)	Neural network (classification for AI-optimized PRS model)	No	10-y risk of MACE (MI, TIA or stroke, and cardiovascular death)
Agrawal et al, 2021^27^	New PRS	UK Biobank (no history of CVD)	Elastic net regression, XGBoost (feature selection+classification for AI-optimized PRS model)	No	10-y risk of CAD

AI = artificial intelligence; CAD = coronary artery disease; LASSO = least absolute shrinkage and selection operator; MACE = major adverse cardiovascular event; MI = myocardial infarction; PGS = polygenic score; PRS = polygenic risk score; TIA = transient ischemic attack; XGBoost = extreme gradient boosting.

### Machine learning models

The studies employed various ML algorithms for feature selection and classification to optimize PRS. Feature selection methods such as elastic net regression (38.5%), random forest (RF) (30.8%), and least absolute shrinkage and selection operator (Lasso) regression (23.1%) were the most commonly used ML approaches, effectively identifying key genetic markers while mitigating overfitting risks (Table 1). For instance, Forrest et al applied RF for both feature selection and classification in an optimized PRS combined with clinical risk factors, achieving 85% accuracy and an area under the curve (AUC) of 0.91 in external validation in the UK Biobank for the prediction of CAD.^19^ Similarly, Alireza et al reported that integrating RF and maximum relevance minimum redundancy (mRMR) for feature selection improved the AUC from 0.54 to 0.80 by incorporating clinical variables such as age, sex, body mass index (BMI), comorbidities, and lipid profiles.^17^ They also indicated that ML algorithms, coupled with effective feature selection techniques, can identify panels of as few as 50 genetic markers, achieving approximately 80% accuracy in predicting CAD when integrated with clinical risk factors.^17^ Neural network algorithms were employed in 3 studies (23.1%).^20^^,^^26^ For example, NeuralCVD, which integrated 29 CVD risk factors with genetic data from 6 additional PRS, demonstrated a 0.006 improvement in the concordance index (C-index) and a net reclassification improvement of 1.16% for predicting 10-year risk of a major adverse cardiovascular event.^26^ This underscores the potential of neural network models to enhance PRS performance and refine clinical risk stratification.

Model performance metrics were inconsistently reported across the included studies. Accuracy was reported in 4 (30.8%) studies, with values ranging from 67% to 89%.^11^^,^^18^, ^19^, ^20^ Confusion matrix-derived metrics, including sensitivity, specificity, and F1-score, were also reported in 4 (30.8%) studies.^11^^,^^18^^,^^19^^,^^23^ Studies by Sng et al and Norland et al reported models with relatively high sensitivity (81% and 76%, respectively), indicating effective case identification.^18^^,^^23^ Sng et al also reported a positive prediction value of 69% and an F1-score of 74% for a model combining PRS, carotid intima-media thickness (CIMT), and conventional risk factors.^18^ Besides, Naderian et al evaluated 3 AI-optimized models combining PRS, polysocial scores, and clinical risk calculators (QRISK3, PREVENT, atherosclerotic cardiovascular disease pooled cohort equations [ASCVD PCE]), which demonstrated high specificity (ranging from 81.0% to 89.3%) and moderate sensitivity (ranging from 39.6% to 57.3%), depending on the actionable threshold applied.^11^ Moreover, Forrest et al reported a model with high sensitivity and specificity in both internal and external validation cohorts, achieving 90% sensitivity and 88% specificity internally, and 84% sensitivity and 83% specificity in external validation (Supplemental Table 1).^19^

### Added features in AI-optimized PRS

Among the 13 included studies, the most prevalent type of added components were clinical risk factors in 10 studies (76.9%).^11^^,^^16^, ^17^, ^18^, ^19^, ^20^^,^^24^, ^25^, ^26^, ^27^ Current evidence suggests that variables such as age, sex, BMI, comorbidities, smoking status, and medications can complement genetic data by accounting for environmental, behavioral, and physiological factors (Table 2).

**Table 2:** AI-Optimized PRS Components, Additional Features, and Main Findings of Included Studies

First Author, Year	AI-Optimized PRS Components	Additional Features Added to PRS	Comparison Parameter	Model Performance (AUC/C-Index)
Naderian et al, 2024^11^	SDOH and lifestyle–psychological factors Clinical risk scores	100 SDOH and lifestyle–psychological factors Clinical risk scores (ASCVD PCE, PREVENT, QRISK3)	C-index, accuracy, sensitivity, specificity	Baseline PRS model (C-index: 0.74) AI-optimized polysocial score model (C-index: 0.75) AI-optimized (combining PRS+polysocial score+PCE clinical score) model (C-index: 0.78) AI-optimized (combining PRS+polysocial score+PREVENT clinical score) model (C-index: 0.77) AI-optimized (combining PRS+polysocial score+QRISK3 clinical score) model (C-index: 0.78)
Mazidi et al, 2024^16^	Proteomics Clinical risk factors	Proteomic data (2,923 plasma proteins) 6 risk factors (age, sex, smoking, DM, SBP, waist circumference)	C-index, net reclassification improvement	Internal validation: Baseline PRS model (C-index: 0.55) Internal validation: Risk factors model (C-index: 0.84) Internal validation: 2,923 proteomic model (C-index: 0.86) Internal validation: AI-optimized 30 proteomic model (C-index: 0.84) Internal validation: AI-optimized (combining PRS+risk factors+2,923 proteomic) model (C-index: 0.87) Internal validation: AI-optimized (combining PRS+risk factors+30 proteomic) model (C-index: 0.86) External validation: Risk factors model (C-index: 0.71) External validation: AI-optimized (combining risk factors+446 proteomic) model (C-index: 0.73)
Alireza et al, 2024^17^	Clinical risk factors	12 risk factors (age, sex, BMI, diabetes, hypertension, vascular/heart problems, smoking, CRP, total cholesterol, triglycerides, HDL, LDL)	AUC	Baseline PRS model (AUC: 0.54) Risk factors model (AUC: 0.76) AI-optimized (combining PRS+12 risk factors) model using GWAS-driven feature selection (AUC: 0.79) AI-optimized (combining PRS+12 risk factors) model using mRMR-based feature selection (AUC: 0.79) AI-optimized (combining PRS+12 risk factors) model using RF-based feature selection (AUC: 0.80)
Sng et al, 2024^18^	Imaging Clinical risk factors	Imaging (carotid intima-media thickness) 4 risk factors (age, sex, BMI, smoking status)	AUC, accuracy, PPV, sensitivity, F1-score	Baseline PRS model (AUC: 0.56) Imaging model (AUC: 0.62) Risk factors model (AUC: 0.84) AI-optimized (combining PRS+imaging+4 risk factors) model (AUC: 0.82)
Forrest et al, 2023^19^	Clinical risk factors	Risk factors (age, sex, BMI, ethnicity, total cholesterol, HDL, SBP, treatment for blood pressure, DM, smoking status)	AUC, accuracy, sensitivity, specificity	AI-optimized (PRS+risk factors) model (AUC: 0.95) Internal validation: AI-optimized (PRS+risk factors) model (AUC: 0.93) External validation: AI-optimized (PRS+risk factors) model (accuracy: 85%, AUC: 0.91)
Klau et al, 2023^20^	Integrating multiple PRS Demographics	139 additional diseases PRS 2 risk factors (age, sex)	AUC, accuracy	Single-PRS model using ridge regression (AUC: 0.79) Single-PRS model using random forest (AUC: 0.77) Single-PRS model using deep neural network (AUC: 0.79) AI-optimized (combining PRS+139 PRS+2 risk factors) model using ridge regression (AUC: 0.79) AI-optimized (combining PRS+139 PRS+2 risk factors) model using random forest (AUC: 0.77) AI-optimized (combining PRS+139 PRS+2 risk factors) model using deep neural network (AUC: 0.79)
Lin et al, 2023^21^	Integrating multiple PRS	10 additional biomarkers and risk factors PRS (HDL, LDL, triglyceride, apolipoprotein A1, apolipoprotein B, creatinine, CRP, HbA1c, cigarettes per day, SBP)	AUC, HR, C-index	Internal validation: Baseline PRS model (HR: 1.62, AUC: 0.79) Internal validation: Biomarkers+10 risk factors PRS model (HR: 1.41, AUC: 0.78) Internal validation: AI-optimized (combining PRS+biomarkers+10 risk factors+CAD PRS) PRS model (HR: 1.72, AUC: 0.80) External validation: Baseline PRS model (HR: 1.57, AUC: 0.75) External validation: Biomarkers+10 risk factors PRS model (HR: 1.27, AUC: 0.73) External validation: AI-optimized (combining PRS+biomarkers+10 risk factors+CAD PRS) PRS model (HR: 1.60, AUC: 0.76)
Møller et al, 2023^22^	Proteomics Imaging	Proteomic data (368 plasma proteins) PROMISE minimal CAD risk score based on imaging data	AUC	Baseline PRS model (AUC: 0.64) Proteomics model (AUC: 0.58) Imaging model (AUC: 0.76) PRS+proteomic model (AUC: 0.66) PRS+imaging model (AUC: 0.79) AI-optimized (combining PRS+proteomics+imaging) model (AUC: 0.80)
Norland et al, 2023^23^	Integrating multiple PRS	AI-optimized PRS model 1: 15 additional risk factors and CVD disease PRS AI-optimized PRS model 2: 115 existing PRS for 75 different traits from the atherosclerosis risk in communities cohort catalog	AUC, OR, PPV, sensitivity	Internal validation: Baseline PRS model (AUC: 0.69) Internal validation: AI-optimized (combining PRS+15 risk factors and CVD PRS) model (AUC: 0.79) Internal validation: AI-optimized (combining PRS+115 PRS) model (AUC: 0.77) External validation: Baseline PRS model (AUC: 0.77) External validation: AI-optimized (combining PRS+15 risk factors and CVD PRS) model (OR: 1.66, AUC: 0.79) External validation: AI-optimized (combining PRS+115 PRS) model (OR: 1.70, AUC: 0.79)
Lu et al, 2022^24^	Integrating multiple PRS Demographics	8 additional PRS (stroke, diabetes, blood pressure, total cholesterol, HDL, LDL, triglyceride, BMI) 2 risk factors (age, sex)	C-index and net reclassification improvement	AI-optimized (combining PRS+8 biomarker and CVD PRS) model (C-index: 0.61) Risk factors model (C-index: 0.71) AI-optimized (combining PRS+8 biomarker and CVD PRS+risk factors) model (C-index: 0.73) AI-optimized (combining PRS+8 biomarker and CVD PRS+China-PAR clinical risk score) model (C-index: 0.77)
Nam et al, 2022^25^	Integrating multiple PRS Demographics	135 diseases PRS 2 risk factors (age, sex)	AUC	Baseline PRS model (AUC: 0.58) Network PRS model (AUC: 0.64) AI-optimized (combining PRS+network PRS+2 risk factors) model (AUC: 0.74)
Steinfeldt et al, 2022^26^	Integrating multiple PRS Clinical risk factors	6 additional PRS 29 risk factors	C-index and net reclassification improvement	Baseline PRS model (C-index: 0.73) Risk factors model (C-index: 0.74) AI-optimized (combining CVD PRS+29 risk factors) model (C-index: 0.75)
Agrawal et al, 2021^27^	Clinical risk factors (waist circumference, socioeconomic deprivation, and 28 biomarkers)	Demographics Anthropometric variables Socioeconomic status 28 biomarkers	C-index and net reclassification improvement	AI-optimized (combining PRS+28 biomarkers+clinical risk factors) model using elastic net regression (C-index: 0.80) AI-optimized (combining PRS+28 biomarkers+clinical risk factors) model using XGBoost (C-index: 0.80)

AUC = area under the curve; ASCVD PCE = atherosclerotic cardiovascular disease pooled cohort equations; BMI = body mass index; CRP = C-reactive protein; CVD = cardiovascular disease; DM = diabetes mellitus; GWAS = genome-wide association study; HBA1C = hemoglobin A1c; HDL = high-density lipoprotein; LDL = low-density lipoprotein; mRMR = maximum relevance minimum redundancy; PCE = pooled cohort equations; PPV = positive predictive value; PREVENT = predicting risk of cardiovascular disease; RF = random forest; SBP = systolic blood pressure; SDOH = social determinants of health; other abbreviations as in Table 1.

Integrating multiple PRS with genetic data for CVD risk factors, biomarkers, and other disease traits has been a key strategy for developing more comprehensive PRS models. Multi-PRS approaches were used in 6 studies (46.2%), leveraging pleiotropy and genetically correlated traits to better capture the multifactorial nature of CVD.^20^^,^^21^^,^^23^, ^24^, ^25^, ^26^ However, the effectiveness of this approach varied among studies, with 3 studies reporting limited gains in predictive power when multiple PRS were combined, particularly when the primary PRS model was generated using ML algorithms.^20^^,^^21^^,^^26^ Additionally, 2 studies emphasized that adding risk factors such as age, sex, and clinical risk scores to multi-PRS models can significantly enhance model performance.^24^^,^^25^ For instance, Lu et al demonstrated that adding age and sex into a multi-PRS model increased the C-index for predicting CAD from 0.61 to 0.73, with further integration of the China-PAR clinical risk score enhanced the C-index to 0.77.^24^ Similarly, Nam et al reported that supplementing a multi-PRS model of genetic data for 135 diseases with age and sex significantly increased the AUC from 0.58 to 0.74 for predicting MI.^25^

Biomarkers, including lipid profiles, diabetes indexes, and inflammatory markers such as C-reactive protein, were incorporated in 3 studies (23.1%) to enhance PRS models by providing molecular-level insights into the pathways involved in CVD.^17^^,^^19^^,^^27^ Integrating these biochemical markers enables PRS models to achieve a more nuanced understanding of disease risk, particularly in relation to metabolic and inflammatory pathways. Imaging data, such as CIMT or computed tomography angiography, were included in 2 studies (15.4%), serving as structural markers of vascular health and disease progression.^18^^,^^22^ For instance, Sng et al combined CIMT data with SNP-based genetic data and traditional risk factors (age, sex, BMI, and smoking status) to predict CAD. The resulting AI-optimized PRS model achieved an accuracy of 72% and an AUC of 0.82, significantly outperforming models based on SNPs alone (accuracy: 56%; AUC: 0.56) or CIMT alone (accuracy: 57%; AUC: 0.62).^18^ These findings emphasize the utility of imaging data in complementing genetic data to improve model predictive performance.

Proteomic data, although used in only 2 studies (15.4%), offer substantial promise for optimizing PRS models by incorporating molecular signatures linked to protein pathways and disease mechanisms.^16^^,^^22^ Møller et al demonstrated that integrating proteomic profiles with PRS and imaging data significantly enhanced model performance, increasing the AUC from 0.64 in the conventional PRS model to 0.80 in the AI-optimized PRS model.^22^ This improvement underscores the value of proteomics in bridging genetic predispositions with functional biological markers, advancing the precision of risk stratification.

### Performance of AI-optimized PRS vs established clinical risk scores

Across all included studies, 6 (46.2%) compared AI-optimized PRS models with established clinical risk scores used in current practice, including ASCVD PCE, QRISK3, Systematic COronary Risk Evaluation 2 (SCORE2), Framingham Risk Score, PREVENT, Polygenic and Multifactorial Risk Score (PMRS), and China-PAR.^11^^,^^21^^,^^22^^,^^24^^,^^26^^,^^27^ Among these, ASCVD PCE and QRISK3 were the most frequently used clinical risk scores, each evaluated in 3 studies.

Improvements in AUC/C-index were reported in 5 studies, ranging from 0.01 to 0.04, suggesting modest gains in discriminatory performance.^11^^,^^21^^,^^22^^,^^26^^,^^27^ In addition, 3 studies reported improvements in net reclassification improvement (NRI), with categorical NRI values ranging from 3.2% to 10.4%, and continuous NRI reaching 15.7%, indicating enhanced risk reclassification over established clinical risk scores.^24^^,^^26^^,^^27^ The comparison of model accuracy, sensitivity, and specificity was reported in only the study by Naderian et al.^11^ This study demonstrated improvements in sensitivity of up to 4.1%, specificity up to 1.2%, and changes in accuracy ranging from −0.3% to +1.3%, depending on the applied threshold and comparator (Supplemental Table 2).

### Risk of bias assessment

**Figure 2:**
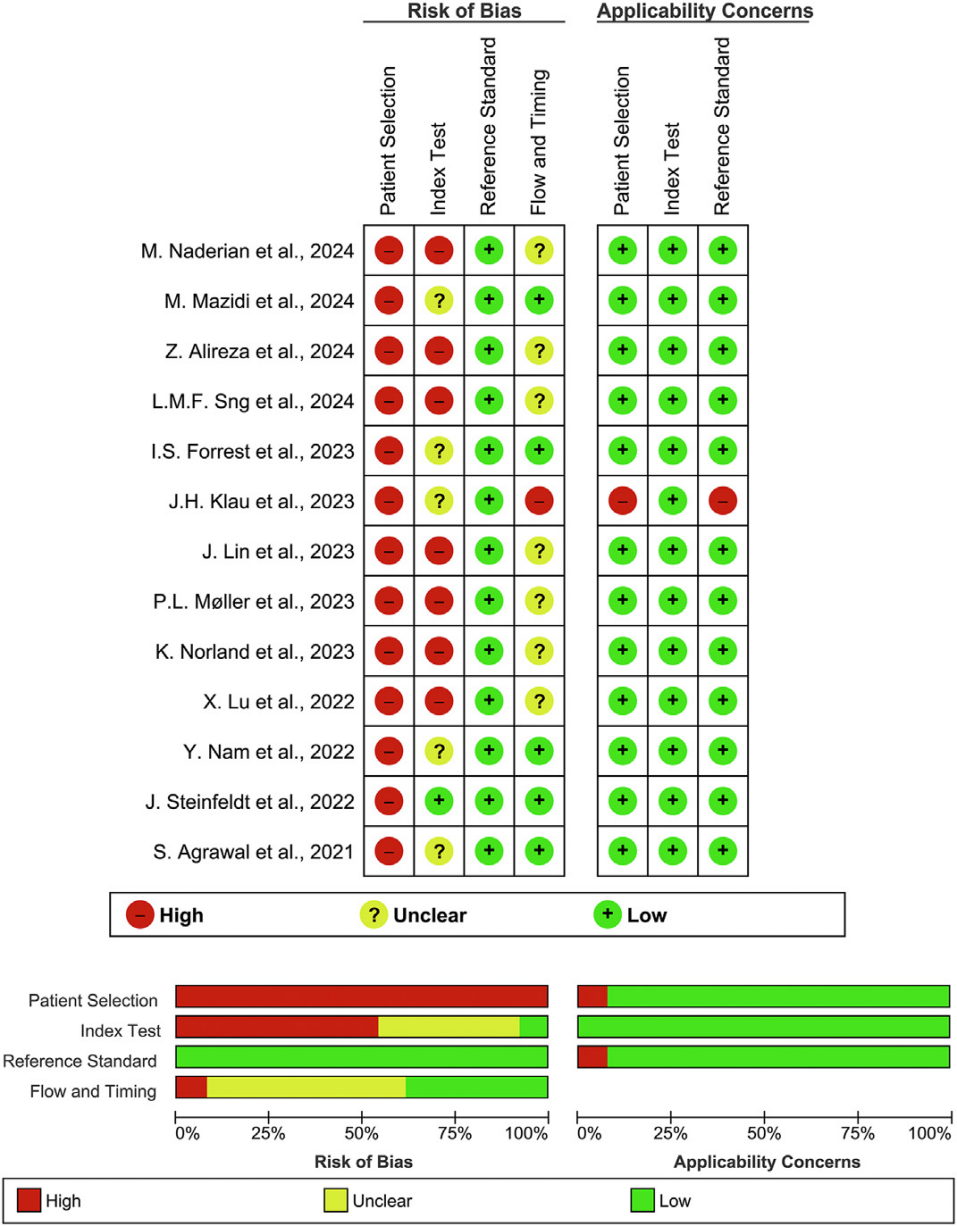
Risk of Bias Assessment Using the QUADAS-2 Tool QUADAS-2 = Quality Assessment of Diagnostic Accuracy Studies-2.

We adapted the QUADAS-2 tool to evaluate the risk of bias and applicability of the included studies. Across all studies, the risk of bias for patient selection was rated as high, primarily due to the reliance on biobank data, which often overrepresented specific ethnic groups and lacked randomization, raising concerns about the representativeness of the study populations. Regarding the index test domain, 46.2% of studies were rated as having a high risk of bias, while 38.5% were rated as unclear. This was largely due to the availability of both data and outcomes within the same biobank, potentially introducing bias and the lack of predefined thresholds for evaluating the index test’s performance in 61.5% of studies. In terms of the reference standard, studies generally demonstrated consistent methods, resulting in a low risk of bias in this domain. However, 53.8% of studies exhibited either an unclear or high risk of bias for the flow and timing domain, primarily due to reliance on EHRs for both predictive model development and the reference standard. The use of International Classification of Diseases, 10th Revision (ICD-10) codes as the reference standard often made it difficult to definitively answer key signaling questions. Applicability concerns were minimal, with 84.6% of studies rated as low concern across all domains, indicating that study populations, index tests, and reference standards were appropriate for the scope of this review. The methodological quality graph and summary of the included studies are presented in Figure 2.

## Discussion

The use of conventional PRS for CVD in clinical practice is rapidly advancing, offering substantial potential to improve risk stratification and preventive care. Beyond CAD, PRS have been utilized to predict the risk of heart failure,^28^ aortic valve stenosis,^29^ spontaneous coronary artery dissection,^30^ and stroke.^31^ Furthermore, integrating PRS with existing cardiovascular risk assessment tools has demonstrated improved predictive accuracy in several studies.^32^^,^^33^ The study by Riveros-McKay et al demonstrated that combining PRS with standard tools, such as pooled cohort equations and QRISK3, led to the reclassification of 10.4% of CAD cases initially categorized as low risk and improved overall risk stratification.^32^ These findings highlight the potential of PRS to refine risk prediction and guide targeted preventive interventions.

However, despite promising results of conventional PRS in CVD risk prediction, several barriers limit their widespread adoption in clinical settings. From an economic perspective, high costs associated with genomic testing and computational infrastructure remain a significant challenge, especially in resource-limited health care environments.^34^^,^^35^ Furthermore, additional limitations include reduced predictive performance in non-European populations due to biases in genomic data sets and the linear aggregation of genetic risks, which oversimplifies the complex interplay between genetic and environmental factors.^6^ Integrating PRS into clinical practice, such as EHRs and decision-support tools, also requires significant investments, specialized clinician training, and a focus on developing more equitable and interpretable PRS frameworks.

From a patient perspective, evidence suggests that PRS insights are generally well-received, with patients valuing the enhanced understanding of their genetic risks and the opportunity for personalized prevention strategies.^36^^,^^37^ A recent randomized controlled trial of 1,018 middle-aged participants with >20% CAD genetic risk, selected from the Estonian Biobank, showed that providing genetic risk information led to notable improvements in both lifestyle and medication adherence.^37^ Over 90% of participants found genetic counseling sessions informative, acknowledging the critical role of both lifestyle modifications and genetic predispositions in managing CVD risk. Physicians in the trial similarly observed positive behavioral changes and strongly supported incorporating genetic risk assessment tools into routine clinical practice, underscoring their feasibility and value in preventive cardiology.

The application of AI and ML algorithms in optimizing PRS has offered great opportunities to enhance model accuracy, reduce data complexity, and integrate data from various sources in CVD risk prediction. ML algorithms can optimize PRS in different domains, including feature selection and identification of the most predictive genetic variants, incorporating biomarkers, imaging, and clinical data, and combining multiple PRS (Central Illustration).

**Central Illustration:**
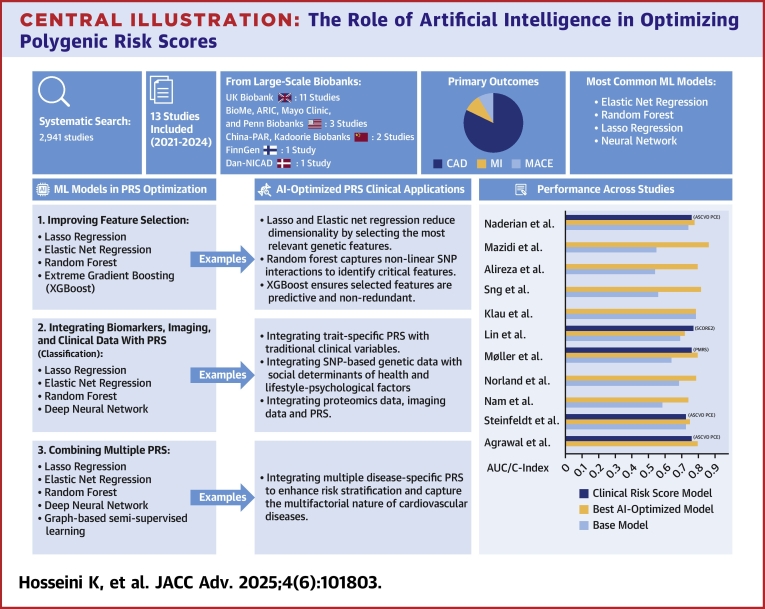
The Role of Artificial Intelligence in Optimizing Polygenic Risk Scores This figure highlights the role of ML algorithms in optimizing PRS for cardiovascular diseases. Data from 9 large biobanks were used to assess primary outcomes, including coronary artery disease (11 studies), myocardial infarction (1 study), and major adverse cardiovascular events (1 study). Key applications of AI tools in PRS optimization are presented: 1) improving feature selection with techniques such as Lasso regression and random forest; 2) integrating PRS with biomarkers, imaging, and clinical variables; and 3) combining multiple PRS using advanced methods like elastic net regression. The figure also compares the predictive performance of the best AI-optimized model with base PRS and clinical risk score models using AUC/C-index as comparison parameters. ASCVD PCE = atherosclerotic cardiovascular disease pooled cohort equation; AUC = area under the curve; CAD = coronary artery disease; MACE = major adverse cardiovascular event; MI = myocardial infarction; ML = machine learning; SNP = single nucleotide polymorphism; XGBoost = extreme gradient boosting; other abbreviations as in Figure 1.

## Improving feature selection

Effective feature selection is a cornerstone of developing robust and clinically applicable PRS. Novel ML algorithms offer distinct advantages in this process, enabling the identification of the most informative genetic variants while mitigating challenges associated with high-dimensional data. Unlike traditional statistical methods, ML techniques can account for complex, nonlinear relationships between genetic variants and cardiovascular outcomes, uncovering patterns that might remain undetected through conventional statistical approaches.^17^^,^^38^^,^^39^ Several ML algorithms have proven particularly useful for feature selection in the genomic context. Models such as Lasso regression, ridge regression, and elastic net regression are among the most commonly used techniques, each incorporating regularization to address the challenges of high-dimensional data sets.^17^ Lasso regression applies L1 regularization, which encourages sparsity by driving some feature coefficients to exactly zero, effectively selecting the most relevant variables. Ridge regression, on the other hand, applies L2 regularization, which penalizes the squared values of coefficients. Unlike Lasso, ridge regression shrinks coefficients toward zero without completely eliminating any features, making it particularly effective for addressing multicollinearity when all variables contribute to the outcome. Elastic net regression combines L1 and L2 regularization, providing a balance between sparsity and robustness. This hybrid approach is especially useful for data sets with correlated predictors, as it accounts for their combined influence more effectively than Lasso or ridge regression alone.^17^^,^^21^^,^^40^

RF and other ensemble learning methods identify feature importance by evaluating how much each feature contributes to reducing the impurity (eg, Gini impurity) or increasing the accuracy of predictions. RF's ability to capture complex, nonlinear interactions between SNPs makes it a powerful tool in genomics, where interdependencies among genetic variants are common.^18^^,^^19^ Furthermore, other algorithms like mRMR focus on selecting features that maximize their relevance to the target variable while minimizing redundancy among the features. This is achieved by using mutual information to measure both relevance and redundancy. By ensuring the features are both highly predictive and minimally correlated, mRMR enhances model interpretability, reduces overfitting risks, and ensures that selected features provide unique, additive information.^38^ The application of these methods enhances conventional feature selection by optimizing the trade-off between model complexity and predictive performance. For instance, Lasso regression can identify sparse feature sets while maintaining high classification accuracy, whereas ensemble methods such as RFs provide robustness by leveraging multiple decision trees and ranking features based on their importance.^17^^,^^39^ These findings highlight the potential benefit of ML in optimizing PRS by enhancing predictive power, simplifying model design, and facilitating more precise and cost-effective clinical applications.

### Improving classification: integrating biomarkers, imaging, and clinical data with PRS

ML algorithms enable the integration of various data sources—biomarkers, imaging data, and clinical risk factors—with PRS to improve the predictive accuracy and robustness of CVD risk prediction models. Recent studies suggest that combining PRS with other data modalities significantly improves model predictive power, enabling a more comprehensive disease risk stratification.^12^^,^^22^^,^^41^, ^42^, ^43^ ML algorithms are particularly effective in handling high-dimensional, heterogeneous data sets, uncovering complex patterns and interactions that traditional statistical methods often overlook. This capability is critical for improving risk stratification and supporting personalized clinical decision-making.

Among ML techniques, Lasso and elastic net regression have proven effective for selecting and regularizing features, allowing the integration of genetic and biomarker data into predictive models while minimizing the risk of overfitting.^12^^,^^22^^,^^44^ RFs and other ensemble learning methods are adept at capturing nonlinear interactions between variables, including SNPs, imaging metrics, and clinical biomarkers.^18^ Additionally, recent advancements in deep learning models—such as convolutional and recurrent neural networks—have demonstrated great potential in combining imaging data with clinical and genetic parameters to further enhance model predictive performance.^22^

Emerging evidence shows that integrating PRS with proteomic signatures, imaging data (eg, CIMT), lifestyle–psychological factors, and cardiovascular risk factors outperforms models relying solely on clinical risk factors.^11^^,^^16^^,^^18^^,^^22^^,^^45^ By leveraging the complementary strengths of PRS and other data types, ML algorithms enable the development of robust, multimodal predictive models, paving the way for the implementation of precision medicine in cardiology practice.

### Improving classification-combining multiple PRS

Integrating multiple PRS offers a powerful strategy for enhancing predictive accuracy and capturing the multifactorial nature of CVD, with ML algorithms playing a key role in this process. Recent studies emphasize the importance of combining PRS derived from genetically correlated CVD traits and risk factors to improve the prediction of CAD.^46^, ^47^, ^48^, ^49^ This approach leverages pleiotropy—a phenomenon where a single genetic variant influences multiple phenotypes—and capitalizes on shared genetic pathways underlying different diseases or traits.^46^ By integrating PRS, multi-PRS models provide a more comprehensive assessment of genetic risk, improving the predictive performance of single-PRS models.^23^

Combining diverse PRS is particularly advantageous for refining CVD risk scores, as it incorporates the genetic architecture of both CVD and its risk factors. For instance, PRS derived from risk factors such as diabetes, hypertension, and hypercholesterolemia, which reflect the genetic contribution to these comorbidities, can be integrated with CVD-specific PRS to further enhance their predictive power.^49^ This strategy has demonstrated significant efficacy in early-onset CAD cases, where genetic factors play a more prominent role, thereby improving risk stratification and guiding timely preventative interventions.^23^

ML techniques such as Lasso and ridge regression are widely employed to optimize multi-PRS models by selecting and weighting the most informative scores, reducing overfitting, and improving model interpretability.^20^^,^^23^ Advanced algorithms, such as RFs and deep neural networks, are particularly adept at capturing nonlinear interactions between PRS and clinical risk factors, enabling the development of more nuanced and data-driven predictive models.^9^^,^^20^^,^^23^ For instance, NeuralCVD—a graph-based semisupervised learning model—integrates 6 PRS with 29 cardiovascular risk factors (including demographic and anthropometric data, comorbidities, blood pressure, and lipid profile), demonstrating superior predictive performance for the 10-year risk of major adverse cardiac events compared with conventional methods.^26^ These advancements highlight how ML algorithms can develop multi-PRS models, leading to more accurate and personalized risk assessments in cardiology.

### AI-optimized PRS clinical applications and advantages

The heterogeneity observed across studies highlights the importance of tailoring PRS models to specific applications and data types. While biomarkers, imaging, and proteomics have shown clear benefits, their impact often depends on the baseline model's characteristics and the ML techniques employed. For instance, studies using baseline PRS models generated by ML algorithms reported smaller incremental gains from adding features, suggesting diminishing returns when the baseline models are already highly predictive.^24^ These findings collectively demonstrate the potential of integrating diverse data types—genetic, clinical, and molecular—to optimize PRS models. Such strategies not only enhance predictive accuracy but also enable more effective risk stratification, guiding personalized prevention and clinical decision-making. By integrating established and emerging data sources, AI-optimized PRS models offer a comprehensive strategy for tackling the multifactorial nature of CVD, positioning them as a transformative tool for future cardiology practice.

The external validation was conducted in only 5 of the 13 included studies (38.5%), highlighting a major limitation in terms of generalizability and clinical applicability. Broader validation in independent, multiethnic cohorts is essential to support the robustness and translational readiness of these models. In addition to limited external validation, the availability and quality of real-world clinical data pose a significant challenge to the adoption of AI-optimized PRS models. Most algorithms are trained and tested in research settings with well-curated data sets, which may not reflect the fragmented or incomplete nature of data in routine clinical environments. Missing genetic, clinical, or biomarker inputs can impair model performance and reduce their practical utility for clinical decision-making.

We must also note that only 30.8% of the included studies reported confusion matrix-derived metrics—such as sensitivity, specificity, and F1-score—and that only Naderian et al explicitly specified the decision threshold used to calculate these metrics.^11^ This lack of standardized reporting, particularly across studies that relied primarily on AUC or C-index, limits comparability and may obscure true model performance, especially in imbalanced data sets where threshold-dependent trade-offs between sensitivity and specificity are critical for clinical implementation. Determining the optimal decision threshold is another key consideration for the model's clinical implementation, as it can directly impact the balance between false positives and false negatives. This choice should be guided by the clinical setting and whether the model is intended for screening (favoring higher sensitivity) or diagnostic confirmation (favoring higher specificity). Eventually, despite promising findings from large national biobank studies, the clinical impact of AI-optimized PRS models remains uncertain. Further randomized controlled trials are needed to evaluate their effectiveness in improving patient outcomes and to support evidence-based implementation, including the adaptation of preventive and therapeutic strategies.

### Ethical, legal, and social implications

The development of AI-optimized PRS for CVD risk prediction presents significant ethical, legal, and social considerations. A major limitation is the limited generalizability of PRS models, as the majority are derived from genomic data sets predominantly representing European populations. This bias reduces predictive accuracy in non-European cohorts, increasing the risk of exacerbating existing health inequities.^50^ It is important to develop and include diverse genomic data sets and implement fairness-aware AI frameworks that account for ancestry-based variations to address this issue.^51^ We also emphasize that generalizability limitations extend beyond genetic ancestry. Predictive models—whether based on genetic, clinical, or biomarker data—often exhibit variable performance across populations due to differences in health care systems, biomarker measurement standards, clinical guidelines, and routine care practices. Addressing these broader sources of heterogeneity is essential for building truly generalizable models.

The integration of PRS with clinical and molecular data, while enhancing predictive performance, raises significant concerns regarding privacy and autonomy. Handling sensitive genomic data, especially when combined with EHRs, demands robust regulatory frameworks and advanced privacy-preserving techniques to mitigate risks of misuse and ensure data security.^50^ Transparent consent processes are also essential to maintaining participant trust and ethical integrity. Furthermore, the implementation of AI-optimized PRS models into clinical workflows may require regulatory classification as medical devices, particularly when algorithms influence clinical decision-making. Such classification necessitates formal assessment of risks and benefits, compliance with regulatory standards, and may impose significant costs related to validation, certification, and postmarket surveillance.

The long-term performance of AI-optimized PRS models requires regular updates to prevent data drift and account for evolving clinical guidelines, diagnostic procedures, and biomarker measurements. Ongoing validation and recalibration are essential to maintain accuracy in real-world settings. In addition, the use of AI in optimizing PRS introduces regulatory and patient engagement complexities. Unlike traditional statistical models, AI-powered tools require extensive individual-level data for training, posing challenges in ensuring secure data sharing, maintaining transparency, and achieving reproducibility.^6^ Current regulatory frameworks often fail to address the dynamic, iterative nature of AI technologies, emphasizing the need for international guidelines that balance innovation with accountability.^52^ Ethical frameworks should also prioritize clear communication of PRS limitations to patients and clinicians, offering guidance on the probabilistic nature of risk predictions. By embedding these considerations into both research and clinical implementation, the field can hopefully enhance equity, trustworthiness, and societal alignment in future cardiology practice.

## Conclusions and future directions

The application of AI and ML algorithms to optimize PRS shows promising potential for improving CVD risk prediction. Our systematic review indicates that AI-optimized PRS models, especially when incorporating clinical risk factors, biomarkers, imaging data, and multiple genetic data from other PRS, may offer incremental improvements over conventional PRS models by capturing more comprehensive individual risk profiles. However, the degree of improvement is variable, and several studies reported only modest gains. Current evidence suggests that these advanced models can better stratify patients, guide personalized prevention strategies, and potentially transform future cardiology practice.

However, several areas require further investigation to better understand the benefits of AI-optimized PRS: First, data on sex differences in genetic risk prediction are limited, and more research is needed to understand how these models perform between men and women, especially given the known sex-specific variation in CVD outcomes.^21^^,^^27^ Second, the inclusion of more diverse populations and various CVD conditions beyond CAD—such as heart failure, arrhythmias, and stroke—is needed to improve the generalizability and applicability of these models. Third, assessing the cost-effectiveness of implementing these models in cardiology practice is also crucial, as they have the potential to optimize resource allocation and reduce health care costs by preventing adverse cardiovascular events through early risk identification. Fourth, methodological heterogeneity and inconsistent reporting across studies make it difficult to determine which features—such as biomarkers, imaging, or social factors—contribute most to predictive improvement, underscoring the need for standardized evaluation frameworks in future. Finally, future research should prioritize assessing the real-world impact of AI-optimized PRS on clinical decision-making and patient outcomes. Addressing these gaps is essential to refine model development and to address the ethical, legal, and social implications associated with their clinical implementation.

## Funding support and author disclosures

The authors have reported that they have no relationships relevant to the contents of this paper to disclose.
