# Supplementary material for: Bridging Genomics to Cardiology Clinical Practice: Artificial Intelligence in Optimizing Polygenic Risk Scores: A Systematic Review
Source: JACC Adv. 2025 Jun 25;4(6):101803. doi: 10.1016/j.jacadv.2025.101803 (PMC12277611; doi:10.1016/j.jacadv.2025.101803)
Supplement: Supplementary Data [file mmc1.pdf]

Bridging Genomics to Cardiology Clinical Practice: Artificial Intelligence in Optimizing Polygenic Risk Scores—A Systematic Review

Supplemental Material

Search Strategy 2

PubMed 2

Embase 3

Scopus 4

The Cochrane Library 5

Total Results...5

Supplemental Tables...6

Supplemental Table 1. Prediction metrics of AI-optimized PRS models............................6

Supplemental Table 2. Comparison between AI-optimized PRS models and clinical risk scores...8

Supplemental Table 3. PRISMA 2020 checklist 11

Search Strategy

PubMed (December 15, 2024)

Search	Query	Results (No.)
#1	"Genetic Risk Score"[Title/Abstract] OR "Polygenic Risk Score"[Title/Abstract] OR "Genomewide Association Study"[Title/Abstract] OR "Polygenic Risk"[Title/Abstract] OR "Genetic Predisposition"[Title/Abstract] OR "GWAS"[Title/Abstract] OR "Genomic Risk"[Title/Abstract] OR "Genomic Risk Score"[Title/Abstract] OR "PRS"[Title/Abstract] OR "Hereditary Risk"[Title/Abstract] OR "Genomic Prediction"[Title/Abstract] OR "Multigenic Risk Score"[Title/Abstract] OR "Polygenic Score"[Title/Abstract] OR "Genomic Profile"[Title/Abstract]	62,709
#2	"Cardiovascular Diseases"[MeSH Terms] OR "Cardiovascular Disease"[Title/Abstract] OR "Coronary Artery Disease"[MeSH Terms] OR "Coronary Artery Disease"[Title/Abstract] OR "Coronary Heart Disease"[Title/Abstract] OR "Ischemic Heart Disease"[Title/Abstract] OR "Angina Pectoris"[MeSH Terms] OR "Angina Pectoris"[Title/Abstract] OR "Myocardial Infarction"[MeSH Terms] OR "Myocardial Infarction"[Title/Abstract] OR "Stroke"[MeSH Terms] OR "Stroke"[Title/Abstract] OR "Atherosclerosis"[MeSH Terms] OR "Atherosclerosis"[Title/Abstract] OR "Heart Attack"[Title/Abstract] OR "Cardiac Infarction"[Title/Abstract] OR "CVD"[Title/Abstract] OR "Cardiac Disease"[Title/Abstract] OR "Cardiac Arrest"[Title/Abstract] OR "Cardiomyopathy"[MeSH Terms] OR "Cardiomyopathy"[Title/Abstract] OR "Heart Failure"[MeSH Terms] OR "Heart Failure"[Title/Abstract] OR "Peripheral Arterial Disease"[MeSH Terms] OR "Peripheral Arterial Disease"[Title/Abstract] OR "Hypertension"[MeSH Terms] OR "Hypertension"[Title/Abstract] OR "Cardiac Ischemia"[Title/Abstract] OR "Vascular Disease"[Title/Abstract]	3,359,386
#3	"Artificial Intelligence"[Title/Abstract] OR "AI"[Title/Abstract] OR "Machine Learning"[Title/Abstract] OR "Deep Learning"[Title/Abstract] OR "Neural Network"[Title/Abstract] OR "Random Forest"[Title/Abstract] OR "Gradient Boosting"[Title/Abstract] OR "Support Vector Machine"[Title/Abstract] OR "Predictive Modeling"[Title/Abstract] OR "Supervised Learning"[Title/Abstract] OR "Unsupervised Learning"[Title/Abstract] OR "Reinforcement Learning"[Title/Abstract] OR "Algorithmic Modeling"[Title/Abstract] OR "Computational Intelligence"[Title/Abstract] OR "Bayesian Networks"[Title/Abstract] OR "Big Data"[Title/Abstract] OR "Data Mining"[Title/Abstract] OR "Predictive Analytics"[Title/Abstract] OR "Decision Trees"[Title/Abstract] or "Cox Model"[Title/Abstract] OR "Proportional Hazards Model"[Title/Abstract] OR "Survival Analysis"[Title/Abstract] OR "Regression Model"[Title/Abstract] OR "Logistic Regression"[Title/Abstract] OR "Elastic Net Regression"[Title/Abstract] OR "Lasso Regression"[Title/Abstract] OR "Ridge Regression"[Title/Abstract] OR "Regularization Methods"[Title/Abstract] OR "Generalized Linear Model"[Title/Abstract] OR "Multivariate Regression"[Title/Abstract] OR "Combined Polygenic Risk Score"[Title/Abstract] OR "Combined PRS"[Title/Abstract]	989,517
#4	#1 AND #2 AND #3	605

Embase (December 15, 2024)

Search	Query	Results (No.)
#1	'Genetic Risk Score':ti,ab OR 'Polygenic Risk Score':ti,ab OR 'Genomewide Association Study':ti,ab OR 'Polygenic Risk':ti,ab OR 'Genetic Predisposition':ti,ab OR 'GWAS':ti,ab OR 'Genomic Risk':ti,ab OR 'Genomic Risk Score':ti,ab OR 'PRS':ti,ab OR 'Hereditary Risk':ti,ab OR 'Genomic Prediction':ti,ab OR 'Multigenic Risk Score':ti,ab OR 'Polygenic Score':ti,ab OR 'Genomic Profile':ti,ab	88,679
#2	'Cardiovascular Disease'/exp OR 'Cardiovascular Disease':ti,ab OR 'Coronary Artery Disease'/exp OR 'Coronary Artery Disease':ti,ab OR 'Coronary Heart Disease':ti,ab OR 'Ischemic Heart Disease':ti,ab OR 'Angina Pectoris'/exp OR 'Angina Pectoris':ti,ab OR 'Myocardial Infarction'/exp OR 'Myocardial Infarction':ti,ab OR 'Stroke'/exp OR 'Stroke':ti,ab OR 'Atherosclerosis'/exp OR 'Atherosclerosis':ti,ab OR 'Heart Attack':ti,ab OR 'Cardiac Infarction':ti,ab OR 'CVD':ti,ab OR 'Cardiac Disease':ti,ab OR 'Cardiac Arrest':ti,ab OR 'Cardiomyopathy'/exp OR 'Cardiomyopathy':ti,ab OR 'Heart Failure'/exp OR 'Heart Failure':ti,ab OR 'Peripheral Arterial Disease'/exp OR 'Peripheral Arterial Disease':ti,ab OR 'Hypertension'/exp OR 'Hypertension':ti,ab OR 'Cardiac Ischemia':ti,ab OR 'Vascular Disease':ti,ab	6,072,799
#3	'Artificial Intelligence':ti,ab OR 'AI':ti,ab OR 'Machine Learning':ti,ab OR 'Deep Learning':ti,ab OR 'Neural Network':ti,ab OR 'Random Forest':ti,ab OR 'Gradient Boosting':ti,ab OR 'Support Vector Machine':ti,ab OR 'Predictive Modeling':ti,ab OR 'Supervised Learning':ti,ab OR 'Unsupervised Learning':ti,ab OR 'Reinforcement Learning':ti,ab OR 'Algorithmic Modeling':ti,ab OR 'Computational Intelligence':ti,ab OR 'Bayesian Networks':ti,ab OR 'Big Data':ti,ab OR 'Data Mining':ti,ab OR 'Predictive Analytics':ti,ab OR 'Decision Trees':ti,ab OR 'Cox Model':ti,ab OR 'Proportional Hazards Model':ti,ab OR 'Survival Analysis':ti,ab OR 'Regression Model':ti,ab OR 'Logistic Regression':ti,ab OR 'Elastic Net Regression':ti,ab OR 'Lasso Regression':ti,ab OR 'Ridge Regression':ti,ab OR 'Regularization Methods':ti,ab OR 'Generalized Linear Model':ti,ab OR 'Multivariate Regression':ti,ab OR 'Combined Polygenic Risk Score':ti,ab OR 'Combined PRS':ti,ab	1,306,244
#4	#1 AND #2 AND #3	1,368

Scopus (December 15, 2024)

Search	Query	Results (No.)
#1	TITLE-ABS("Genetic Risk Score") OR TITLE-ABS("Polygenic Risk Score") OR TITLE-ABS("Genomewide Association Study") OR TITLE-ABS("Polygenic Risk") OR TITLE-ABS("Genetic Predisposition") OR TITLE-ABS("GWAS") OR TITLE-ABS("Genomic Risk") OR TITLE-ABS("Genomic Risk Score") OR TITLE-ABS("PRS") OR TITLE-ABS("Hereditary Risk") OR TITLE-ABS("Genomic Prediction") OR TITLE-ABS("Multigenic Risk Score") OR TITLE-ABS("Polygenic Score") OR TITLE-ABS("Genomic Profile")	74,211
#2	TITLE-ABS("Cardiovascular Disease") OR TITLE-ABS("Coronary Artery Disease") OR TITLE-ABS("Coronary Heart Disease") OR TITLE-ABS("Ischemic Heart Disease") OR TITLE-ABS("Angina Pectoris") OR TITLE-ABS("Myocardial Infarction") OR TITLE-ABS("Stroke") OR TITLE-ABS("Atherosclerosis") OR TITLE-ABS("Heart Attack") OR TITLE-ABS("Cardiac Infarction") OR TITLE-ABS("CVD") OR TITLE-ABS("Cardiac Disease") OR TITLE-ABS("Cardiac Arrest") OR TITLE-ABS("Cardiomyopathy") OR TITLE-ABS("Heart Failure") OR TITLE-ABS("Peripheral Arterial Disease") OR TITLE-ABS("Hypertension") OR TITLE-ABS("Cardiac Ischemia") OR TITLE-ABS("Vascular Disease")	2,051,385
#3	TITLE-ABS('Artificial Intelligence') OR TITLE-ABS('AI') OR TITLE-ABS('Machine Learning') OR TITLE-ABS('Deep Learning') OR TITLE-ABS('Neural Network') OR TITLE-ABS('Random Forest') OR TITLE-ABS('Gradient Boosting') OR TITLE-ABS('Support Vector Machine') OR TITLE-ABS('Predictive Modeling') OR TITLE-ABS('Supervised Learning') OR TITLE-ABS('Unsupervised Learning') OR TITLE-ABS('Reinforcement Learning') OR TITLE-ABS('Algorithmic Modeling') OR TITLE-ABS('Computational Intelligence') OR TITLE-ABS('Bayesian Networks') OR TITLE-ABS('Big Data') OR TITLE-ABS('Data Mining') OR TITLE-ABS('Predictive Analytics') OR TITLE-ABS('Decision Trees') OR TITLE-ABS('Cox Model') OR TITLE-ABS('Proportional Hazards Model') OR TITLE-ABS('Survival Analysis') OR TITLE-ABS('Regression Model') OR TITLE-ABS('Logistic Regression') OR TITLE-ABS('Elastic Net Regression') OR TITLE-ABS('Lasso Regression') OR TITLE-ABS('Ridge Regression') OR TITLE-ABS('Regularization Methods') OR TITLE-ABS('Generalized Linear Model') OR TITLE-ABS('Multivariate Regression') OR TITLE-ABS('Combined Polygenic Risk Score') OR TITLE-ABS('Combined PRS')	4,418,280
#4	#1 AND #2 AND #3	935

The Cochrane Library (December 15, 2024)

Search	Query	Results (No.)
#1	"Genetic Risk Score":ti,ab OR "Polygenic Risk Score":ti,ab OR "Genomewide Association Study":ti,ab OR "Polygenic Risk":ti,ab OR "Genetic Predisposition":ti,ab OR "GWAS":ti,ab OR "Genomic Risk":ti,ab OR "Genomic Risk Score":ti,ab OR "PRS":ti,ab OR "Hereditary Risk":ti,ab OR "Genomic Prediction":ti,ab OR "Multigenic Risk Score":ti,ab OR "Polygenic Score":ti,ab OR "Genomic Profile":ti,ab	3,029
#2	"Cardiovascular Disease":ti,ab OR "Coronary Artery Disease":ti,ab OR "Coronary Heart Disease":ti,ab OR "Ischemic Heart Disease":ti,ab OR "Angina Pectoris":ti,ab OR "Myocardial Infarction":ti,ab OR "Stroke":ti,ab OR "Atherosclerosis":ti,ab OR "Heart Attack":ti,ab OR "Cardiac Infarction":ti,ab OR "CVD":ti,ab OR "Cardiac Disease":ti,ab OR "Cardiac Arrest":ti,ab OR "Cardiomyopathy":ti,ab OR "Heart Failure":ti,ab OR "Peripheral Arterial Disease":ti,ab OR "Hypertension":ti,ab OR "Cardiac Ischemia":ti,ab OR "Vascular Disease":ti,ab	220,783
#3	"Artificial Intelligence":ti,ab OR "AI":ti,ab OR "Machine Learning":ti,ab OR "Deep Learning":ti,ab OR "Neural Network":ti,ab OR "Random Forest":ti,ab OR "Gradient Boosting":ti,ab OR "Support Vector Machine":ti,ab OR "Predictive Modeling":ti,ab OR "Supervised Learning":ti,ab OR "Unsupervised Learning":ti,ab OR "Reinforcement Learning":ti,ab OR "Algorithmic Modeling":ti,ab OR "Computational Intelligence":ti,ab OR "Bayesian Networks":ti,ab OR "Big Data":ti,ab OR "Data Mining":ti,ab OR "Predictive Analytics":ti,ab OR "Decision Trees":ti,ab OR "Cox Model":ti,ab OR "Proportional Hazards Model":ti,ab OR "Survival Analysis":ti,ab OR "Regression Model":ti,ab OR "Logistic Regression":ti,ab OR "Elastic Net Regression":ti,ab OR "Lasso Regression":ti,ab OR "Ridge Regression":ti,ab OR "Regularization Methods":ti,ab OR "Generalized Linear Model":ti,ab OR "Multivariate Regression":ti,ab OR "Combined Polygenic Risk Score":ti,ab OR "Combined PRS":ti,ab	52,283
#4	#1 AND #2 AND #3	33

Total Results (December 15, 2024)

**Database**	**Results (No.)**
PubMed	605
Embase	1,368
Scopus	935
The Cochrane Library	33
**Total**	**2,941**

Supplemental Tables

Supplemental Table 1. Prediction metrics of AI-optimized polygenic risk score models in included studies

First author, year	Accuracy	PPV/Sensitivity/Specificity/F1-score
M. Naderian et al., 2024[11]	- AI-optimized (combining PRS+polysocial score+QRISK3 clinical score) model:<br>• Actionable threshold of 7.5% (Accuracy: 80.1%)<br>• Actionable threshold of 10% (Accuracy: 86.4%)<br><br>- AI-optimized (combining PRS+polysocial score+PREVENT clinical score) model:<br>• Actionable threshold of 7.5% (Accuracy: 79.8%)<br>• Actionable threshold of 10% (Accuracy: 86.9%)<br><br>- AI-optimized (combining PRS+polysocial score+PCE clinical score) model:<br>• Actionable threshold of 7.5% (Accuracy: 80.2%)<br>• Actionable threshold of 10% (Accuracy: 86.7%)	- AI-optimized (combining PRS+polysocial score+QRISK3 clinical score) model:<br>• Actionable threshold of 7.5% (Sensitivity: 57.3%, Specificity: 81.3%)<br>• Actionable threshold of 10% (Sensitivity: 42.8%, Specificity: 88.7%)<br><br>- AI-optimized (combining PRS+polysocial score+PREVENT clinical score) model:<br>• Actionable threshold of 7.5% (Sensitivity: 55.1%, Specificity: 81.0%)<br>• Actionable threshold of 10% (Sensitivity: 39.6%, Specificity: 89.3%)<br><br>- AI-optimized (combining PRS+polysocial score+PCE clinical score) model:<br>• Actionable threshold of 7.5% (Sensitivity: 56.4%, Specificity: 81.4%)<br>• Actionable threshold of 10% (Sensitivity: 42.3%, Specificity: 89.0%)
M. Mazidi et al., 2024[16]	—	—
Z. Alireza et al., 2024[17]	—	—
L.M.F. Sng et al., 2024[18]	- Base PRS model (Accuracy: 56%)<br>- Imaging model (Accuracy: 57%)<br>- Traditional Risk factors model (Accuracy: 77%)<br>- Optimized (PRS+imaging+risk factors) model (Accuracy: 72%)	- Base PRS model (PPV: 55%, Sensitivity: 64%, F1-score: 59%)<br>- Imaging model (PPV: 57%, Sensitivity: 62%, F1-score: 60%)<br>- Traditional Risk factors model (PPV: 79%, Sensitivity: 73%, F1-score: 76%)<br>- Optimized (PRS+imaging+risk factors) model (PPV: 69%, Sensitivity: 81%, F1-score: 74%)
I.S. Forrest et al., 2023[19]	- Optimized (PRS+risk factors) model (accuracy: 88%)<br>- Internal validation: Optimized (PRS+risk factors) model (accuracy: 89%)<br>- External validation: Optimized (PRS+risk factors) model (accuracy:85%)	- Optimized (PRS+risk factors) model (Sensitivity: 94%, Specificity: 82%)<br>- Internal validation: Optimized (PRS+risk factors) model (Sensitivity: 90%, Specificity: 88%)<br>- External validation: Optimized (PRS+risk factors) model (Sensitivity: 84%, Specificity: 83%)

First author, year	Accuracy	PPV/Sensitivity/Specificity/F1-score
J.H. Klau et al., 2023[20]	- Single-PRS model using ridge regression (Accuracy: 70%)<br>- Single-PRS model using random forest (Accuracy: 67%)<br>- Single-PRS model using deep neural network (Accuracy: 69%)<br>- Optimized (PRS+risk factors) model using ridge regression (Accuracy: 69%)<br>- Optimized (PRS+risk factors) model using random forest (Accuracy: 68%)<br>- Optimized (PRS+risk factors) model using deep neural network (Accuracy: 70%)	—
J. Lin et al., 2023[21]	—	—
P.L. Møller et al., 2023[22]	—	—
K. Norland et al., 2023[23]	—	- External validation: Base PRS model (PPV: 21%, Sensitivity: 72%)<br>- External validation: Optimized (Combining PRS+15 risk factors and CVD PRSs) model (PPV: 21%, Sensitivity: 75%)<br>- External validation: Optimized (Combining PRS+115 PRSs) model (PPV: 21%, Sensitivity: 76%)
X. Lu et al., 2022[24]	—	—
Y. Nam et al., 2022[25]	—	—
J. Steinfeldt et al., 2022[26]	—	—
S. Agrawal et al., 2021[27]	—	—

Abbreviations: PPV: positive predictive value.

Supplemental Table 2. Comparison between AI-optimized polygenic risk score models and clinical risk scores in included studies

First author, year	Clinical Risk Scores	Comparison Between AI-Optimized PRS vs. Clinical Risk Scores
M. Naderian et al., 2024[11]	QRISK3, PREVENT, ASCVD PCE	**• Predicting CAD (AI-optimized [PRS+polysocial score+QRISK3] model vs. QRISK3):**<br>- Improvement in C-index: 0.02<br>- Actionable threshold of 7.5%, improvement in Accuracy: 0.6%, Sensitivity: 3.0%, Specificity: 0.5%<br>- Actionable threshold of 10%, improvement in Accuracy: -0.3%, Sensitivity: 4.1%, Specificity: -0.5%<br><br>**• Predicting CAD (AI-optimized [PRS+polysocial score+PREVENT] model vs. PREVENT):**<br>- Improvement in C-index: 0.03<br>- Actionable threshold of 7.5%, improvement in Accuracy: 1.1%, Sensitivity: 1.5%, Specificity: 1.1%<br>- Actionable threshold of 10%, improvement in Accuracy: 0.4%, Sensitivity: 2.9%, Specificity: 0.3%<br><br>**• Predicting CAD (AI-optimized [PRS+polysocial score+ASCVD PCE] model vs. ASCVD PCE):**<br>- Improvement in C-index: 0.02<br>- Actionable threshold of 7.5%, improvement in Accuracy: 1.3%, Sensitivity: 1.9%, Specificity: 1.2%<br>- Actionable threshold of 10%, improvement in Accuracy: 0.7%, Sensitivity: 3.2%, Specificity: 0.6%
M. Mazidi et al., 2024[16]	—	—
Z. Alireza et al., 2024[17]	—	—
L.M.F. Sng et al., 2024[18]	—	—
I.S. Forrest et al., 2023[19]	—	—
J.H. Klau et al., 2023[20]	—	—

First author, year	Clinical Risk Scores	Comparison Between AI-Optimized PRS vs. Clinical Risk Scores
J. Lin et al., 2023[21]	SCORE2	**• Predicting CAD in internal validation (UK Biobank):**<br>- Improvement in C-index: 0.02<br>- Optimized PRS model: HR: 1.72 (95% CI: 1.67–1.77)<br>- SCORE2: HR: 1.37 (95% CI: 1.34–1.40)<br><br>**• Predicting CAD in external validation (Finngen Biobank):**<br>- Improvement in C-index: -0.05<br>- Optimized PRS model: HR: 1.60 (95% CI: 1.58–1.62)<br>- SCORE2: HR: 1.34 (95% CI: 1.28–1.41)
P.L. Møller et al., 2023[22]	PMRS	**• Predicting CAD:**<br>- Improvement in AUC: 0.04
K. Norland et al., 2023[23]	—	—
X. Lu et al., 2022[24]	China-PAR*	**• Predicting CAD (Optimized PRS model vs. China-PAR):**<br>- Categorical net reclassification improvement [cut-off ≥4.5%]: 3.2 (95% CI: 0.9-5.8)<br>- Continuous net reclassification improvement: 15.7 (95% CI: 7.7-22.2)
Y. Nam et al., 2022[25]	—	—
J. Steinfeldt et al., 2022[26]	SCORE, ASCVD PCE, QRISK3	**• Predicting MACE (Optimized PRS model vs. SCORE):**<br>- Improvement in C-index: 0.037 (95%CI: 0.034-0.039)<br>- Categorical net reclassification improvement [cut-off: 10%]: 0.104 (95% CI: 0.098-0.110)<br><br>**• Predicting MACE (Optimized PRS model vs. ASCVD PCE):**<br>- Improvement in C-index: 0.024 (95%CI: 0.023-0.026)<br>- Categorical net reclassification improvement [cut-off: 10%]: 0.070 (95% CI: 0.065-0.076)<br><br>**• Predicting MACE (Optimized PRS model vs. QRISK3):**<br>- Improvement in C-index: 0.010 (95%CI: 0.009-0.011)<br>- Categorical net reclassification improvement [cut-off: 10%]: 0.049 (95% CI: 0.044-0.053)

First author, year	Clinical Risk Scores	Comparison Between AI-Optimized PRS vs. Clinical Risk Scores
S. Agrawal et al., 2021[27]	Framingham Risk Score, ASCVD PCE, QRISK3	**• Predicting CAD (Optimized PRS model vs. Framingham Risk Score):**<br>- Improvement in C-index: 0.040<br>- Categorical net reclassification improvement [cut-off: 2.5%]: 0.060 (95% CI: 0.035-0.086)<br>- Categorical net reclassification improvement [cut-off: 5.0%]: 0.061 (95% CI: 0.031-0.091)<br><br>**• Predicting CAD (Optimized PRS model vs. ASCVD PCE):**<br>- Improvement in C-index: 0.042<br>- Categorical net reclassification improvement [cut-off: 2.5%]: 0.066 (95% CI: 0.041-0.091)<br>- Categorical net reclassification improvement [cut-off: 5.0%]: 0.082 (95% CI: 0.051-0.112)<br><br>**• Predicting CAD (Optimized PRS model vs. QRISK3):**<br>- Improvement in C-index: 0.035<br>- Categorical net reclassification improvement [cut-off: 2.5%]: 0.058 (95% CI: 0.033-0.083)<br>- Categorical net reclassification improvement [cut-off: 5.0%]: 0.075 (95% CI: 0.046-0.105)

* The China-PAR risk of 4.5% for 10-year CAD risk is equivalent to the ASCVD PCE risk of 10%.

Abbreviations: ASCVD: atherosclerotic cardiovascular disease, AUC: area under the curve, CAD: coronary artery disease, China-PAR: prediction for atherosclerotic cardiovascular disease risk in China, CI: confidence interval, HR: hazard ratio, MACE: major adverse cardiovascular event, PCE: pooled cohort equations, PMRS: prospective multicenter imaging study for evaluation of chest pain minimal risk score, PPV: positive predictive value, PREVENT: predicting risk of cardiovascular disease EVENTs, SCORE: systematic coronary risk evaluation.

Supplemental Table 3. PRISMA 2020 checklist

Section and Topic	Item #	Checklist item	Location where item is reported
TITLE			
Title	1	Identify the report as a systematic review, meta-analysis, or both.	Page 1, *Title*
ABSTRACT			
Abstract	2	Provide a structured summary including, as applicable: background; objectives; data sources; study eligibility criteria, participants, and interventions; study appraisal and synthesis methods; results; limitations; conclusions and implications of key findings; systematic review registration number.	Page 2, *Abstract*
INTRODUCTION			
Rationale	3	Describe the rationale for the review in the context of existing knowledge.	Page 5, lines 93-109
Objectives	4	Provide an explicit statement of questions being addressed with reference to participants, interventions, comparisons, outcomes, and study design (PICOS).	Page 5, lines 109-111
METHODS			
Protocol and registration	5	Indicate if a review protocol exists, if and where it can be accessed (e.g., Web address), and, if available, provide registration information including registration number.	Page 6, lines 114-116
Eligibility criteria	6	Specify the inclusion and exclusion criteria for the review and how studies were grouped for the syntheses.	Page 6, lines 123-129
Information sources	7	Describe all information sources (e.g., databases with dates of coverage, contact with study authors to identify additional studies) in the search and date last searched.	Pages 6, lines 119-123
Search strategy	8	Present full electronic search strategy for at least one database, including any limits used, such that it could be repeated.	Supplemental Material
Selection process	9	Specify the methods used to decide whether a study met the inclusion criteria of the review, including how many reviewers screened each record and each report retrieved, whether they worked independently, and if applicable, details of automation tools used in the process.	Page 6, lines 130-132

Section and Topic	Item #	Checklist item	Location where item is reported
Data collection process	10	Specify the methods used to collect data from reports, including how many reviewers collected data from each report, whether they worked independently, any processes for obtaining or confirming data from study investigators, and if applicable, details of automation tools used in the process.	Page 6, lines 130-132
Data items	11a	List and define all outcomes for which data were sought. Specify whether all results that were compatible with each outcome domain in each study were sought (e.g. for all measures, time points, analyses), and if not, the methods used to decide which results to collect.	Pages 6-7, lines 132-135
	11b	List and define all other variables for which data were sought (e.g. participant and intervention characteristics, funding sources). Describe any assumptions made about any missing or unclear information.	Pages 6-7, lines 132-135
Study risk of bias assessment	12	Specify the methods used to assess risk of bias in the included studies, including details of the tool(s) used, how many reviewers assessed each study and whether they worked independently, and if applicable, details of automation tools used in the process.	Page 7, lines 136-148
Effect measures	13	Specify for each outcome the effect measure(s) (e.g. risk ratio, mean difference) used in the synthesis or presentation of results.	Not Applicable
Synthesis methods	14	Describe the methods of handling data and combining results of studies, if done, including measures of consistency (e.g., I^2) for each meta-analysis.	Not Applicable
Reporting bias assessment	15	Specify any assessment of risk of bias that may affect the cumulative evidence (e.g., publication bias, selective reporting within studies).	Not Applicable
Additional analyses	16	Describe methods of additional analyses (e.g., sensitivity or subgroup analyses, meta-regression), if done, indicating which were pre-specified.	Not Applicable
RESULTS			
Study selection	17	Give the number of studies screened, assessed for eligibility, and included in the review, with reasons for exclusions at each stage, ideally with a flow diagram.	Page 7, lines 151-153
Study characteristics	18	Cite each included study and present its characteristics.	Pages 7-8, lines 153-161

Section and Topic	Item #	Checklist item	Location where item is reported
Risk of bias in studies	19	Present assessments of risk of bias for each included study.	Page 12, lines 247-264 and Figure 2
Results of individual studies	20	For all outcomes, present, for each study: (a) summary statistics for each group (where appropriate) and (b) an effect estimate and its precision (e.g. confidence/credible interval), ideally using structured tables or plots.	Pages 8-12, lines 162-246 and Table 2, Supplemental Tables 1 and 2
Results of syntheses	21	Present results of each meta-analysis done, including confidence intervals and measures of consistency.	Not Applicable
Reporting biases	22	Present assessments of risk of bias due to missing results (arising from reporting biases) for each synthesis assessed.	Not Applicable
Additional analysis	23	Give results of additional analyses, if done (e.g., sensitivity or subgroup analyses, meta-regression [see Item 16]).	Not Applicable
DISCUSSION			
Discussion	24a	Provide a general interpretation of the results in the context of other evidence.	Pages 13-14, lines 267-300
	24b	Discuss any limitations of the evidence included in the review.	Pages 18-19, lines 389-424
	24c	Discuss any limitations of the review processes used.	Pages 19-21, lines 426-460
	24d	Discuss the implications of the results for practice, policy, and future research.	Pages 21-22, lines 463-486
OTHER INFORMATION			
Support	25	Describe sources of financial or non-financial support for the review, and the role of the funders or sponsors in the review.	Page 1, lines 27-28
Competing interests	26	Declare any competing interests of review authors.	Page 1, lines 24-25
Availability of data, code, and other materials	27	Report which of the following are publicly available and where they can be found: template data collection forms; data extracted from included studies; data used for all analyses; analytic code; any other materials used in the review.	Page 22, lines 488-490
